# Early prediction of language and literacy problems: is 18 months too early?

**DOI:** 10.7717/peerj.1098

**Published:** 2015-07-23

**Authors:** Fiona J. Duff, Kate Nation, Kim Plunkett, DVM Bishop

**Affiliations:** Department of Experimental Psychology, University of Oxford, UK

**Keywords:** Late talkers, Specific language impairment, Language, Literacy

## Abstract

There is a lack of stability in language difficulties across early childhood: most late talkers (LTs) resolve their difficulties by pre-school; and a significant number of children who were not LTs subsequently manifest language difficulties. Greater reliability in predicting individual outcomes is needed, which might be achieved by waiting until later in development when language is more stable. At 18 months, productive vocabulary scores on the Oxford Communicative Developmental Inventory were used to classify children as LTs or average talkers (ATs). Thirty matched-pairs of LTs and ATs were followed up at school-age (average age 7 years), when language and literacy outcomes were assessed. For 18 children, intermediate testing at age 4 had classified them as showing typical development (TD) or specific language impairment (SLI). After correcting for multiple comparisons, there were no significant differences between the LTs and ATs on any outcome measure, and the LTs were performing in the average range. However, there were large-sized effects on all outcomes when comparing the TD and SLI groups. LT status on its own is not determinative of language and literacy difficulties. It would therefore not be appropriate to use expressive vocabulary measures alone to screen for language difficulties at 18 months. However, children with language impairment at age 4 are at risk of enduring difficulties.

The term ‘late talkers’ (LTs) generally refers to young children aged 18- to 35-months who are slow to develop spoken language in the absence of any known primary cause (Rescorla, 2011). Prevalence rates for LTs differ according to the inclusion criteria and the population sampled, but a recent study of a large community sample suggested that as many as 20% of young children can be classified as LTs (Reilly et al., 2007). A key question is whether LTs should be a cause for concern; here there can be a mismatch between views of academic researchers and those concerned with policy.

Research evidence shows that there is a lack of stability in language across early childhood development—particularly when measured by vocabulary—making prediction of outcomes from infancy unreliable. For example, while the early language difficulties of some LTs persist into childhood, the majority of LTs perform in the average range by pre-school (Rescorla, 2011). Moreover, a significant number of children show late emerging language delay: that is, children who were not originally classified as LTs go on to exhibit language difficulties in the pre-school years (e.g., Dale et al., 2003; Henrichs et al., 2011). It is also important to consider the longer-term outcomes of LTs, given the proposal that children whose language skills appear to have normalised will in fact manifest language and literacy difficulties later in development—so called illusory recovery (Scarborough & Dobrich, 1990; but see Dale et al., 2014). In fact, the majority of LTs perform in the average range on language and literacy measures in the later primary school years and beyond, though often at a level significantly below that of their unaffected peers (e.g., Dale & Hayiou-Thomas, 2013; Paul et al., 1997; Rescorla, 2002; Rescorla, 2005; Rescorla, 2009; Rice, Taylor & Zubrick, 2008). In the main, then, the language difficulties of most LTs are short-lived.

Nevertheless, outside of academia there are those who claim that LTs should be identified and supported early on. The logic is that by intervening early, there is a better chance of avoiding the worst outcomes and of making effective changes while the brain is still plastic. This viewpoint is articulated by the C4EO Early Intervention Expert Group (2010), who note that many children with language delay catch up with their peers, but assert that this is because they have had “the right support.” This is rather misleading, since most studies report good outcomes despite lack of intervention. This is a critical point, because if there is spontaneous improvement in LTs, then early intervention is not warranted, especially if it takes funds from other deserving causes. Unfortunately, spontaneous improvement can also reinforce the misapprehension surrounding outcomes of LTs: if these children are identified and provided with intervention, they then improve, and the improvement is attributed to the intervention. The lack of treatment effect is only evident if one studies an untreated group of LTs, as was done in the study by Wake et al. (2011). Nevertheless, the view remains among some of those influencing policy that a child’s vocabulary level at or before 2 years of age can be used to predict language and pre-literacy skills at school entry (e.g., Roulstone et al., 2011).

An important goal, then, is to be able to distinguish better between early language difficulties that will resolve versus persist – that is, to improve prediction of outcomes at an individual level. This might be achieved by waiting until later in development, once language may have become more stable, or by identifying better predictors early on. Here, we take the former approach. We report a follow-up investigation of a sample of children who were identified as LTs at 18 months old, and of their peers who were classed as average talkers. Our first aim was to compare the language and literacy outcomes of these groups of children. Based on previous findings, we hypothesised that the LTs’ school-age language and literacy skills would be in the average range, but fall significantly below those of their unaffected peers. Information regarding language status at age 4 (typical development vs. specific language impairment) was available for a subsample of children, allowing us to explore a second hypothesis that language status at 4 years would be more indicative of reading and language outcomes than language status at 18 months.

## Method

### Participants

Children in the present study were all part of a broader research programme investigating language and literacy development (Duff et al., 2015). Informed written parental consent was given for all participants, and ethical approval was granted by the University of Oxford’s Central University Research Ethics Committee (MSD/IDREC/C1/2012/56). For the main project, 300 children whose vocabularies had been measured in infancy (between 16 and 24 months) were followed-up at school-age, when they were aged between 4 and 9 years old. Though age was allowed to vary at both test points, it was accounted for in the statistical analyses.

In the present report, we followed earlier investigations (e.g., Bishop et al., 2012) by focusing on those children whose vocabulary was measured at 18–19 months (*N* = 153). These children were subsequently classified either as ‘late talkers’ (LTs) or ‘average talkers’ (ATs). Classification procedures followed that of Bishop et al. (2012) and used data from the *Oxford Communicative Development Inventory* (*OCDI*; Hamilton, Plunkett & Schafer, 2000). This Anglicised adaptation of the American CDI (Fenson et al., 1994) comprises 416 words, and parents were required to indicate which of these words their child was able to understand (comprehension) and understand and say (production). The first 12 items, which are instances of onomatopoeia, were excluded from analyses leaving a total of 404 items. Children were categorised as LTs if their raw OCDI productive vocabulary score at 18 or 19 months of age (*t*1) was 10 words or fewer. In the sample used by Bishop et al. (2012) this equated to performance levels at least 1 SD below the mean (see https://osf.io/t35af/). However, for the current, slightly younger sample, this cut-off corresponded to the 25th centile for the norms of Hamilton, Plunkett & Schafer (2000), whereas a cut-off of six words or less corresponded to the 16th centile (i.e., equivalent to one SD below the mean). We used the more liberal criterion of 10 words or fewer for this study, but in the analysis, we consider the extent to which the inclusion of children with only mild delays affects the findings. Thirty (22 boys) of the 153 children were classified as LTs (20%) using the 10-word cut-off, and twenty of these met the more stringent cut-off of six words or fewer. Following Bishop et al. (2012), ATs were those children whose OCDI production raw scores fell between 14 and 65 out of 404; this corresponds to the 31st to 73rd centile on the norms for 18- to 19-month-olds from Hamilton, Plunkett & Schafer (2000). This yielded 66 children, of whom 30 were matched pairwise to the LTs, based on gender and age at *t*2 (average absolute difference in age = 1.33 months, range = 0–3 months).

OCDI scores at *t*1 are reported in Table 1, both for the full sample of 30 pairs, and for the 20 pairs with a child meeting the more severe cut-off of 6 words or fewer. Paired-samples *t*-tests revealed that the groups did not differ on vocabulary comprehension; although there was a trend for lower comprehension when a stricter cut-off for LTs was used. Note, however, that the mean comprehension scores were virtually identical for the 20 strictly-defined LTs and the 30 selected by the laxer criterion. Demographic information at *t*1 and *t*2 is also given in Table 1. Children ranged in age from 4 to 9 years at *t*2, but owing to the matching procedure, there was no group-level difference in age. The Index of Multiple Deprivation (IMD) was calculated based on postcode data to give an indication of socioeconomic status (SES). The IMD returns rank-ordered data, ranging from 1 (high deprivation) to 32,482 (low deprivation). Both groups have IMD scores higher than the national average (16,241) but similar to the average for their local county of Oxfordshire (21,809) (Department for Communities and Local Government, 2011). IMD did not differ significantly between the two groups at either time point.

**Table 1:** Comparison of average talkers (ATs) and late talkers (LTs) on vocabulary knowledge at *t*1, and demographic factors at *t*1 and *t*2, with strict and lax definition of LTs.

Measure	LT definition	*N*	AT mean (SD)	LT mean (SD)	*t*	*p*	*d*
OCDI Comprehension *t*1	≤10 words	30	174.7 (65.74)	147.0 (7.72)	1.38	.178	0.41
	≤6 words	20	190.6 (61.65)	147.1 (65.29)	1.87	.077	0.68
OCDI Production *t*1^a^	≤10 words	30	34.77 (13.38)	5.20 (3.21)	–	–	3.04
	≤6 words	20	33.30 (11.55)	3.45 (2.33)	–	–	3.58
Age *t*1 (months)	≤10 words	30	18.30 (0.47)	18.23 (0.43)	0.70	.489	0.16
	≤6 words	20	18.25 (0.44)	18.15 (0.37)	1.00	.330	0.25
IMD Index *t*1	≤10 words	26	24,900 (4,911)	24,985 (6,181)	−0.06	.954	−0.02
	≤6 words	17	24,914 (5,309)	25,692 (4,805)	−0.62	.546	−0.15
Age *t*2 (years; months)^b^	≤10 words	30	7;01 (1;05)	7;01 (1;04)	–	–	0.02
	≤6 words	20	6;11 (1;03)	6;11 (1;04)	–	–	0.01
IMD Index *t*2	≤10 words	27	24,288 (5,910)	22,117 (7,779)	1.12	.272	0.31
	≤6 words	18	24,620 (6,388)	23,322 (7,584)	0.59	.566	0.19

**Notes.**

a Variable used to define non-overlapping LT and AT groups.

b Variable used to match groups.

Of the 30 matched AT/LT pairs in the present study, 9 had previously been assessed at age 4 as part of a separate study by Bishop et al. (2012). At that time point, children were categorised according to whether or not they reached criteria for Specific Language Impairment (SLI). Full details are given in Bishop et al. (2012). Briefly, children were identified as having SLI if their performance was impaired on at least two language measures, but their nonverbal IQ was in the average range (≥85). Children were classified as having typical development (TD) if no more than one language measure was impaired, and their nonverbal IQ was in the average range. In both the AT and LT groups, 3 children were classified as having SLI and 6 children as TD. ^1^1The rate of ATs with SLI in the current study is higher than expected, possibly due to sampling bias, if parents whose children had language problems were more willing to consent to the follow-up. In the original study with a larger sample, the rates were 29% of LTs and 14% of ATs (see Bishop et al., 2012).

### Measures

#### Vocabulary knowledge

The *Receptive* and *Expressive One Word Picture Vocabulary Tests* (Brownell, 2000) were administered. To assess receptive vocabulary, children heard a series of graded words, and were asked to select the corresponding picture from four alternatives for each word (test/re-test reliability = .78 to .93). For expressive vocabulary, children were required to name a series of graded pictures (test/re-test reliability = .88 to .91).

#### Phonological short-term memory

Measures of nonword and sentence repetition tapped short-term memory for verbal information. On the *Children’s Test of Nonword Repetition* (Gathercole & Baddeley, 1996), children repeated aloud 40 individual nonwords, ranging from two to five syllables in length (test/re-test reliability = .72). For the *Recalling Sentences* subtest of the *Clinical Evaluation of Language Fundamentals* (*CELF-III UK*–Semel, Wiig & Secord, 2000), children were required to repeat orally presented sentences of increasing length and grammatical complexity (test/re-test reliability = .93 to .94).

#### Phonological awareness

The *Elision* subtest of the *Comprehensive Test of Phonological Processing* (Wagner, Torgesen & Rashotte, 1999) was administered. For each orally presented word, children were asked to delete a sublexical unit (syllable or phoneme) and supply the word that remained (test/re-test reliability = .79 to .88).

#### Reading accuracy

For the *Diagnostic Test of Word Reading Processes* (Forum for Research into Language and Literacy, 2012), children read aloud lists of graded nonwords, regular words and exception words (reliability, *α* = .99).

#### Reading comprehension

Passage reading comprehension was assessed in children aged 5 upwards via the *York Assessment of Reading Comprehension* (Snowling et al., 2009). Children read aloud two short stories and after each story answered a series of eight related questions (reliability, *α* = .48 to .77).

#### Nonverbal ability

Nonverbal reasoning was assessed via the *Matrices* subtest of the *British Abilities Scale II* (Elliot, Smith & McCulloch, 1997). Children were presented with an incomplete matrix of abstract figures and were instructed to choose the correct shape from an array of six to complete the matrix (test/re-test reliability = .64).

### Procedure

For the follow-up assessments at *t*2, children were seen individually by a member of the research team. Assessment sessions lasted approximately 1 h and were conducted at school, home, or the Department of Experimental Psychology, University of Oxford.

## Results

The scores for the ATs and LTs at follow-up (*t*2) are reported in Table 2. The standardised scores show that the AT group is performing in the average to above average range on all outcomes. For the full sample of 60 children, the maximum number achieving scores below average (>*t*1 SD below the normative mean) on any given measure is 4 (13%). The LT group performed in the average to high-average range on all outcomes, with no more than 3 children (10%) achieving below average scores on any one measure. For statistical analysis, we used raw scores with age regressed out of them (on the basis of the entire dataset of *N* = 300 from Duff et al., 2015). This gives scores that are highly correlated with the standardised scores but with greater precision. (To facilitate readability of Table 2, these means are not included; the data are available in Supplemental Information).

**Table 2:** Language, literacy and nonverbal measures at *t*2 for full sample of Average Talkers (ATs) and Late Talkers (LTs), with strict and lax definitions of LT.

Measure	LT definition	*N*	AT mean (SD)	LT mean (SD)	*t*	*p*	*d*
Receptive vocabulary^a^	≤10 words	30	116.77 (11.15)	112.97 (12.01)	1.33	.194	0.33
	≤6 words	20	117.95 (11.50)	107.25 (16.07)	1.41	.176	0.50
Expressive vocabulary^a^	≤10 words	30	113.67 (13.01)	105.60 (16.18)	2.13	.042	0.58
	≤6 words	20	119.95 (9.60)	114.85 (12.77)	2.36	.029	0.82
Nonword repetition^a^	≤10 words	30	116.38 (17.43)	111.54 (15.23)	1.24	.224	0.33
	≤6 words	18	119.44 (15.43)	112.50 (15.59)	1.57	.132	0.57
Recalling sentences^b^	≤10 words	26	10.33 (2.00)	9.89 (2.68)	1.02	.315	0.27
	≤6 words	18	10.89 (1.94)	9.94 (2.84)	1.79	.091	0.58
Phonological elision^b^	≤10 words	30	10.92 (2.38)	11.46 (3.00)	−0.52	.605	−0.14
	≤6 words	18	10.78 (2.69)	11.89 (3.12)	−0.60	.555	−0.22
Reading accuracy^a^	≤10 words	27	109.70 (16.16)	108.04 (15.84)	0.71	.482	0.17
	≤6 words	18	111.94 (16.56)	109.56 (14.89)	0.80	.434	0.27
Reading comprehension^a^	≤10 words	27	114.08 (7.10)	111.84 (9.22)	0.95	.352	0.25
	≤6 words	16	115.69 (5.76)	112.94 (9.45)	1.28	.221	0.46
Nonverbal IQ^c^	≤10 words	27	55.15 (8.19)	56.74 (10.28)	−0.46	.651	−0.13
	≤6 words	18	54.83 (7.70)	55.17 (11.07)	−0.12	.909	−0.04

**Notes.**

Standardised score means shown here to allow comparison with norms; *t*-tests were performed on age-residualised raw scores (see text).

a Standardised scores are standard scores (*M* = 100, SD = 15).

b Standardised scores are scaled scores (*M* = 10, SD = 3).

c Standardised scores are *T* scores (*M* = 50, SD = 10).

There is a trend for the LTs to have lower scores on most measures (apart from phonological elision and nonverbal IQ). Matched-pairs *t*-tests were performed on the age-regressed scores to assess whether there were any significant differences between the groups. Effect sizes (Cohen’s *d*) were calculated for each contrast by dividing the difference in group means by the pooled standard deviation; *d*s of 0.2, 0.5 and 0.8 represent small, medium and large effects, respectively (Cohen, 1992). Concerning the sample of 30 LTs and their matched ATs, there was a significant medium-sized effect on expressive vocabulary. However, after correcting for multiple comparisons using the Benjamini–Hochberg procedure (Benjamini & Hochberg, 1995), this difference was no longer significant. There was no effect of group on any of the remaining language, literacy, or nonverbal measures.

All analyses were repeated using just the 20 LTs with OCDI production scores of 6 words or fewer, and their matched ATs. As can be seen in Table 2, this tended to give greater effect sizes, but did not have a material effect on the pattern of results. Once again, the only difference reaching the .05 level of significance was on expressive vocabulary, and this did not survive Benjamini–Hochberg correction for multiple comparisons. The similarity of results with the two cut-offs suggests that the severity of the initial expressive language delay is not related to the extent of language deficit at *t*2. To check this impression further, Pearson correlations were computed between the *t*1 OCDI production score of the LT member of a pair and the difference between pair members on each of the *t*2 variables from Table 2. None of the correlations was significant at the .05 level.

We turn now to the subsample of 9 AT/LT matched pairs who had previously been assessed at age 4. At *t*2 in the present study, they were on average aged 8 years, 9 months (range = 8;01 to 9;04). Contrasts between this subsample of ATs and LTs were similar to those for the whole sample: there were no significant differences (all *p*s >.20).

Table 3 shows how LI status at 4 years relates to outcomes at *t*2. The TD group performed in the average to above average range on all outcomes, while the SLI group performed in the below average to high-average range. Comparison of age-regressed scores across the two groups revealed large-sized effects on all outcome measures, favouring the TD group. According to independent samples *t*-tests, the group effect was significant (even after correcting for multiple comparisons) for expressive vocabulary, nonword repetition, recalling sentences, reading accuracy and reading comprehension. The group effect was not significant for receptive vocabulary, phonological elision, or nonverbal IQ.

**Table 3:** Language, literacy and nonverbal scores at *t*2 for the subsample, grouped by language status at 4 years of age (Typical Development (TD) vs. Specific Language Impairment (SLI)).

Measure	Group	*N*	Standardised score (SD)	*t*	*p*	*d*
Receptive vocabulary^a^	TD	12	119.17 (11.50)	2.03	.060	1.04
	SLI	6	109.00 (7.38)			
Expressive vocabulary^a^	TD	12	116.42 (7.70)	5.06	<.001	2.30
	SLI	6	96.67 (9.46)			
Nonword repetition^a^	TD	12	120.38 (7.50)	4.22	.001	1.95
	SLI	6	81.75 (15.44)			
Recalling sentences^b^	TD	12	10.08 (1.08)	4.45	<.001	2.24
	SLI	6	7.67 (1.21)			
Phonological elision	TD	12	12.00 (2.14)	1.82	.088	0.92
	SLI	6	8.67 (1.86)			
Reading accuracy^a^	TD	12	117.67 (8.16)	5.24	<.001	2.19
	SLI	6	91.50 (13.13)			
Reading comprehension^a^	TD	12	115.75 (5.38)	3.48	.003	1.71
	SLI	6	107.67 (6.28)			
Nonverbal IQ^c^	TD	12	57.92 (6.26)	2.01	.062	0.94
	SLI	6	51.50 (7.56)			

**Notes.**

Raw scores were corrected for age at *t*2; *t*-tests were performed on the age-regressed scores.

a Standardised scores are standard scores (*M* = 100, SD = 15).

b Standardised scores are scaled scores (*M* = 10, SD = 3).

c Standardised scores are *T* scores (*M* = 50, SD = 10).

## Discussion

We investigated the school-age outcomes of a group of children defined as LTs at age 18 months, a subsample of whom had also been assessed for SLI at age 4 years. This enabled us to test the hypotheses that the subsequent language and literacy skills of LTs would be in the average range for their age, but fall below the level of their unaffected peers (ATs); and that language status at 4 years would be more indicative of outcomes than language status at 18 months.

Regarding the first hypothesis, the LT group performed comfortably in the average range on all language, literacy and nonverbal measures—with very few individuals reaching criterion for an impairment. In fact, there were no statistically significant differences between the LTs and ATs on any of the outcomes, and all contrasts reflected small effect sizes—except on expressive vocabulary where there was a medium-sized effect in favour of the ATs. Overall, then, we found no evidence for subclinical problems in this group of LTs. We considered whether this null result might be due to use of a lax cut-off for LTs of 10 words or fewer on OCDI Production. However, results were virtually identical when analysis was confined to the 20 LTs with more serious expressive delays, with six words or fewer at *t*1. Furthermore, the severity of vocabulary delay at *t*1 was unrelated to the size of difference between LTs and their matched AT controls at *t*2. Note, however, that we did not include a measure of grammatical ability at *t*2; thus, it remains possible that weaknesses may have been detected in this area of language.

Turning to the second hypothesis, results from our subsample of children showed that, at a group level, while LT status at 18 months did not differentiate language and literacy outcomes at 7 years of age, SLI status at 4 years did. Children with SLI went on to have lower scores on all outcome measures compared to the TD children, and the magnitude of the differences reflected large-sized effects. Despite the low power from the small sample size, differences were statistically significant for expressive vocabulary, nonword repetition, recalling sentences, reading accuracy and reading comprehension (but not receptive vocabulary, phonological elision, or nonverbal IQ). Moreover, the differences were educationally significant, with the SLI subgroup performing 20 standard score points below the TD subgroup on expressive vocabulary and 26 points below on reading accuracy.

Our findings have added to the literature which shows that LT status on its own, defined on the basis of parent-reported expressive vocabulary, is by no means determinative of language and literacy difficulties (e.g., Dale & Hayiou-Thomas, 2013; Paul et al., 1997), that parent report of expressive vocabulary in infancy is not a reliable indicator of outcomes (e.g., Dale et al., 2003), and that language skills—as measured by vocabulary—are not stable across infancy into childhood (e.g., Duff et al., 2015; Ghassabian et al., 2013; Reilly et al., 2010). It follows that it would not be appropriate to use expressive vocabulary measures alone to screen for language difficulties in infancy. Our results also suggest that presence of a language impairment at age 4 years is a much better indicator of enduring difficulties than being an LT at age 18 months. In any time series, one expects to see stronger correlations between adjacent time points than between more remote points, and to some extent this may account for the better prediction of outcome from later ages. However, this cannot explain why prediction is better from, say, 4 to 5 years, as opposed to prediction from 2 to 3 years. To account for that, it seems necessary to invoke the idea that whatever causes persistent language impairment can be distinguished from the factors determining the age at which the child starts to rapidly acquire words. There appears to be a wide range of normal variation in the latter process which can be seen as part of maturation rather than reflective of any disorder. The older a child is, the lower the probability that poor language is just due to normal maturational variation.

A question of interest concerns the optimal age to identify children at risk for persistent language difficulties: given that prediction is poor at 18 months and good at 4 years, we may ask whether there is a step change in predictive utility of language assessment. Dollaghan & Campbell (2009) found that children with a vocabulary deficit at 4 years had a significantly increased risk for manifesting a vocabulary deficit at 6 years, while a deficit at 3 years was not associated with a later impairment. This provides some indication that prediction of outcomes improves after age 3; however, the authors were cautious about their results, as a slight change to the criterion for a vocabulary deficit rendered the elevated risk of persistent deficits from 4 to 6 years nonsignificant. A recent study by Dale & Hayiou-Thomas (2013) showed that the odds of having a language or literacy difficulty at age 12 were higher for LTs identified at age 3 rather than at age 2. However, these difficulties were still only apparent in a minority of children, and the ability to predict which of the LTs at age 3 would subsequently manifest an impairment was poor. This suggests that even prediction of outcomes from age 3 is not sufficiently reliable (see also Zambrana et al., 2014).

It is important to stress that the results of the current study are based on a small sample of children who are not fully representative of the population in terms of SES. Furthermore, the LT group was selected on the basis of expressive vocabulary, without regard to language comprehension (in-keeping with the classic definition of LTs). In the full sample there was a non-significant trend for the LTs to have lower vocabulary comprehension at 18 months. It is likely that a delay in early vocabulary development might assume more importance in children whose development is compromised by other risk factors. Various risks have been shown to be additive to that associated with late-talking, for example, male gender, receptive language difficulties, and family history of language or literacy difficulties (e.g., Law et al., 2012; Ghassabian et al., 2013; Reilly et al., 2010). Nevertheless, to date, models that incorporate such variables have failed to explain enough variance in language outcomes to be usefully predictive at an individual level. A major goal for future research is to generate models that can discriminate reliably between transient versus persistent early language delay, and these models must be simple enough to be useful clinically. Our study, however, suggests that a more fruitful approach may be to conduct longitudinal studies that measure language from infancy into the later primary years, perhaps annually, to determine at what point in development language becomes stable enough for reliable prognoses to be made.

## Supplemental Information

10.7717/peerj.1098/supp-1 Supplemental Information 1 Data for full sample (for analyses reported in Tables 1 and 2) and sub sample (for analyses reported in Table 3)

## Additional Information and Declarations

### Data Deposition

The following information was supplied regarding the deposition of related data: Open Science Framework: https://osf.io/t35af/.
